# Stereotactic Body Radiation Therapy for Patients with Pulmonary Interstitial Change: High Incidence of Fatal Radiation Pneumonitis in a Retrospective Multi-Institutional Study

**DOI:** 10.3390/cancers10080257

**Published:** 2018-08-02

**Authors:** Hiroshi Onishi, Hideomi Yamashita, Yoshiyuki Shioyama, Yasuo Matsumoto, Kenji Takayama, Yukinori Matsuo, Akifumi Miyakawa, Haruo Matsushita, Masahiko Aoki, Keiji Nihei, Tomoki Kimura, Hiromichi Ishiyama, Naoya Murakami, Kensei Nakata, Atsuya Takeda, Takashi Uno, Takuma Nomiya, Tuyoshi Takanaka, Yuji Seo, Takafumi Komiyama, Kan Marino, Shinichi Aoki, Ryo Saito, Masayuki Araya, Yoshiyasu Maehata, Licht Tominaga, Kengo Kuriyama

**Affiliations:** 1 Department of Radiology, School of Medicine, University of Yamanashi, 1110 Shimokato, Chuo, Yamanashi 4093898, Japan; takafumi@yamanashi.ac.jp (T.K.); marino@yamanashi.ac.jp (K.M.); aokis@yamanashi.ac.jp (S.A.); rsaito@yamanashi.ac.jp (R.S.); ai.65718@ai-hosp.or.jp (M.A.); maehata.ro@gmail.com (Y.M.); lichtt@gmail.com (L.T.); kurikuriayumi@gmail.com (K.K.); 2 Department of Radiology, University of Tokyo Hospital, 7-3-1 Hongo, Bunkyo-ku, Tokyo 1138655, Japan; yamachan07291973@yahoo.co.jp; 3 Ion Beam Therapy Center, SAGA-HIMAT Foundation, 3049 Harukoga-machi, Tosu-shi, Saga 8410071, Japan; yshioyama@gmail.com; 4 Department of Radiation Oncology, Niigata Cancer Center Hospital, 2-15-3 Kawagishi, Chuo-ku, Niigata 9518566, Japan; ymatsu@niigata-cc.jp; 5 Department of Radiation Oncology and Image-Applied Therapy, Graduate School of Medicine, Kyoto University, 54 Kawaharacho, Shogoin, Sakyo-ku, Kyoto 6068507, Japan; tkym@kuhp.kyoto-u.ac.jp (K.T.); ymatsuo@kuhp.kyoto-u.ac.jp (Y.M.); 6 Department of Radiology, School of Medicine, Nagoya City University, 1 Kawasumi, Mizuho-cho, Mizuho-ku, Nagoya 4678601, Japan; netakky115@hotmail.com; 7 Department of Radiation Oncology, School of Medicine, Tohoku University, 2-1 Seiryo-machi, Aoba-ku, Sendai, Miyagi 9808574, Japan; harumatsu2001@yahoo.co.jp; 8 Department of Radiology and Radiation Oncology, Hirosaki University School of Medicine, 5 Zaifu-cho Hirosaki city, Aomori 0368562, Japan; maoki@hirosaki-u.ac.jp; 9 Department of Radiation Oncology, Tokyo Metropolitan Cancer and Infectious Diseases Center Komagome Hospital, 3-18-22 Honkomagome, Bunkyo-ku, Tokyo 1138677, Japan; knihei.cick@gmail.com; 10 Department of Radiation Oncology, Hiroshima University Hospital, 1-2-3, Kasumi Minami-ku, Hiroshima 7348551, Japan; tkkimura@hiroshima-u.ac.jp; 11 Department of Radiation Oncology, Kitasato University School of Medicine, 1-15-1 Kitasato, Minami, Sagamihara, Kanagawa 2520375, Japan; hishiyam@kitasato-u.ac.jp; 12 Department of Radiation Oncology, National Cancer Center Hospital, 5-1-1 Tsukiji, Chuo-ku, Tokyo 1040045, Japan; namuraka@ncc.go.jp; 13 Department of Radiation Oncology, Sapporo Medical University, 16-291 Minami-1jyo-nishi, Chuo-ku, Sapporo-shi, Hokkaido 0608543, Japan; kensei@sapmed.ac.jp; 14 Radiation Oncology Center, Ofuna Chuo Hospital, 6-2-24 Ofuna, Kamakura, Kanagawa 2470056, Japan; takedaatsuya@gmail.com; 15 Diagnostic Radiology and Radiation Oncology, Graduate School of Medicine, Chiba University, 1-8-1 Inohana, Chuo-ku, Chiba City, Chiba 2608670, Japan; unotakas@faculty.chiba-u.jp; 16 Department of Radiation Oncology, Yamagata University Faculty of Medicine, 2-2-2 Iida-Nishi, Yamagata-shi, Yamagata 9909585, Japan; t.nomiya@med.id.yamagata-u.ac.jp; 17 Department of Radiation Oncology, Kanazawa University, 13-1 Takaramachi, Kanazawa 9208641, Japan; takanaka@staff.kanazawa-u.ac.jp; 18 Department of Radiation Oncology, Osaka University Graduate School of Medicine, 2-2 (D10) Yamada-oka, Suita, Osaka 5650871, Japan; seo@radonc.med.osaka-u.ac.jp

**Keywords:** stereotactic body radiation therapy, lung cancer, pulmonary interstitial change, radiation pneumonitis

## Abstract

Pretreatment pulmonary interstitial change (PIC) has been indicated as a risk factor of severe radiation pneumonitis (RP) following stereotactic body radiation therapy (SBRT) for early-stage lung cancer, but details of its true effect remain unclear. This study aims to evaluate treatment outcomes of SBRT for stage I non-small cell lung cancer in patients with PIC. A total of 242 patients are included in this study (88% male). The median age is 77 years (range, 55–92 years). A total dose of 40–70 Gy is administered in 4 to 10 fractions during a 4-to-25 day period. One, two, and three-year overall survival (OS) rates are 82.1%, 57.1%, and 42.6%, respectively. Fatal RP is identified in 6.9% of all patients. The percent vital capacity <70%, mean percentage normal lung volume receiving more than 20 Gy (>10%), performance status of 2–4, presence of squamous cell carcinoma, clinical T2 stage, regular use of steroid before SBRT, and percentage predicting forced expiratory volume in one second (<70%) are associated with worse prognoses for OS. Our results indicate that fatal RP frequently occurs after SBRT for stage I lung cancer in patients with PIC.

## 1. Introduction

Hypofractionated stereotactic body radiation therapy (SBRT) delivers a high-dose concentration to tumor with limited toxicity to normal structures. Due to this advantage, SBRT is commonly performed in patients with small-sized primary or oligometastatic lung tumors as a radical and minimally invasive treatment; it is also utilized in patients who are medically inoperable due to poor pulmonary function with chronic lung disease. Excellent outcomes of SBRT were recently reported for medically operable patients with stage I non-small cell lung cancer (NSCLC) [1,2]. However, although SBRT is generally safe, and in most patients, has no severe adverse effects, fatal radiation pneumonitis (RP) can occasionally occur. Fatal RP shows various patterns of inflammatory shadows inside or outside of the irradiated volume, and accompanied by pulmonary edema or infection, may rapidly progress to death. Therefore, identification of risk factors after SBRT is important for identifying and managing patients who are at risk for developing RP. These risk factors, however, have not been clarified. Pretreatment pulmonary interstitial change (PIC) has been indicated as a major risk factor for severe RP after SBRT for early stage lung cancer or pulmonary oligometastases [3,4,5], but the number of patients in each of these studies was small and the details of their background remain unclear. Therefore, the purpose of this study was to evaluate the treatment outcomes and determine risk factors for fatal RP after SBRT for stage I NSCLC in patients with PIC in a large database of a Japanese multi-institutional study group.

## 2. Results

**Figure 1:**
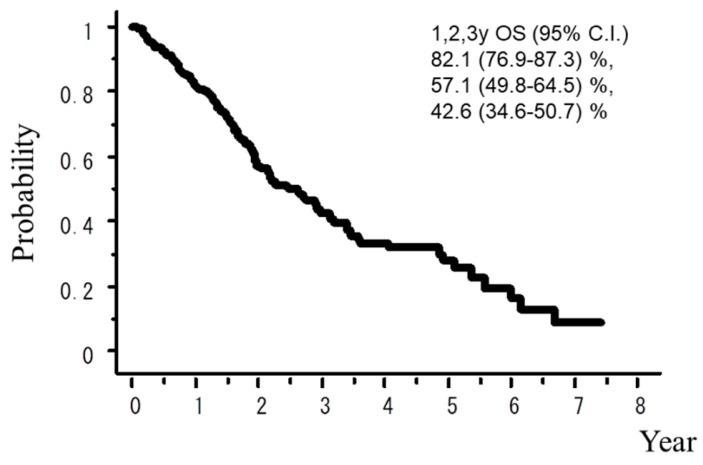
Overall survival (OS) rate.

**Figure 2:**
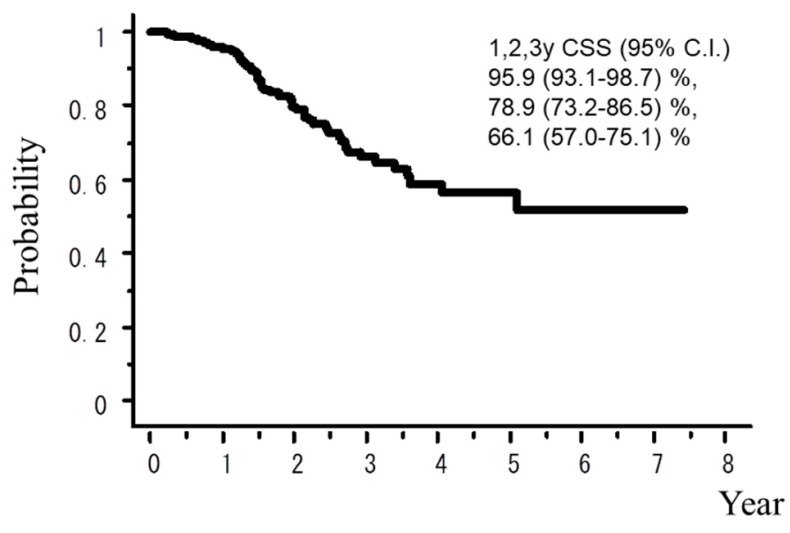
Cause-specific survival rate.

**Figure 3:**
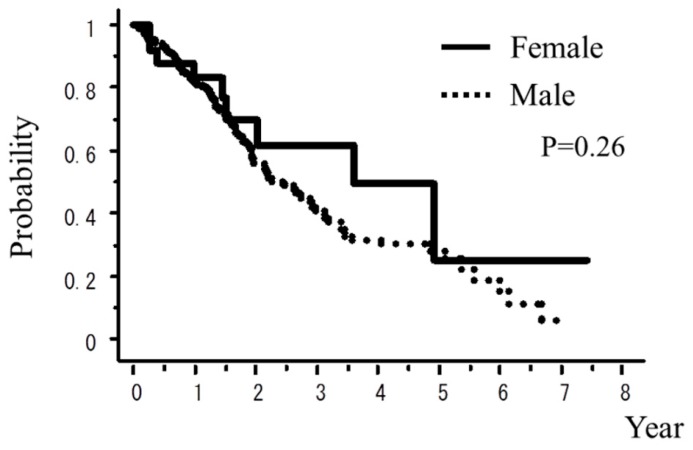
OS rate according to gender.

**Figure 4:**
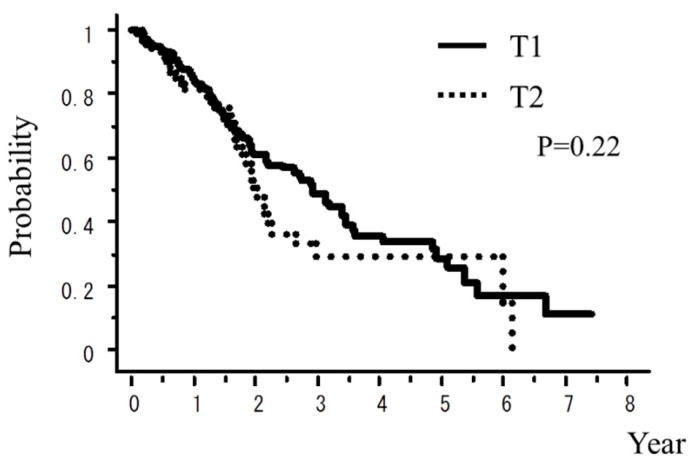
OS rate according to T stage.

**Figure 5:**
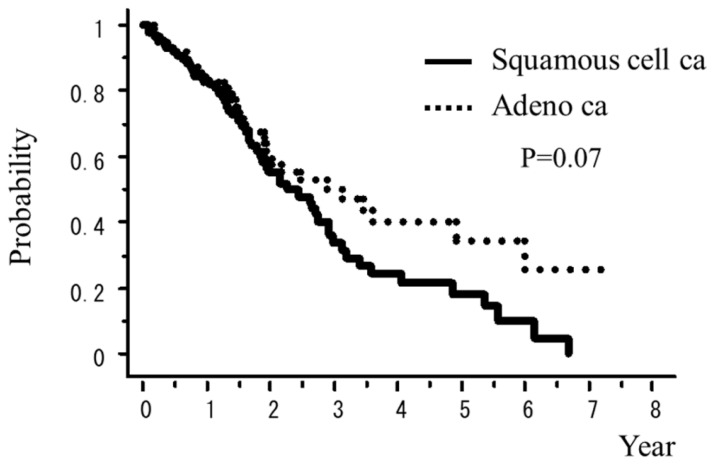
OS rate according to pathology (adeno ca vs squamous cell ca.).

**Figure 6:**
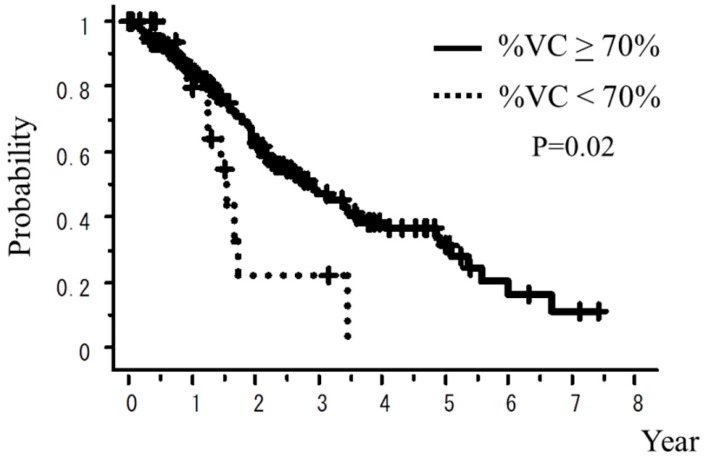
OS rate according to % VC.

**Figure 7:**
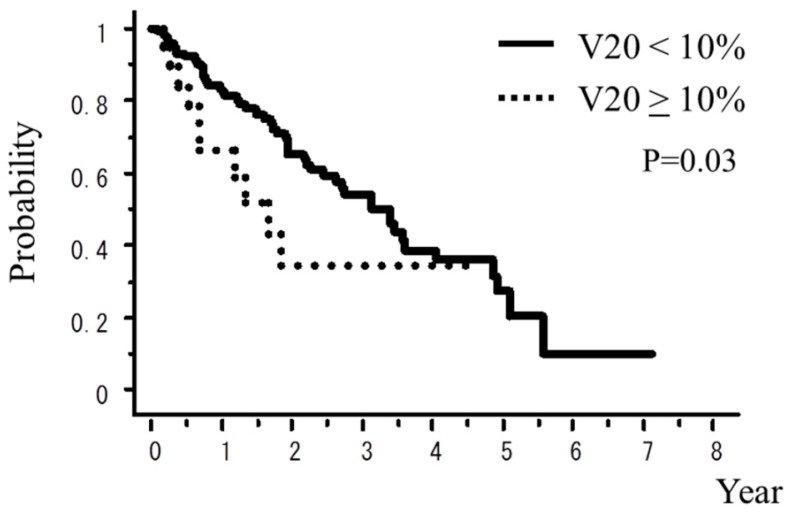
OS rate according to V20 (the mean percentage of normal lung volume receiving more than 20 Gy).

A total of 242 patients in the multi-institutional SBRT study group in Japanese Radiological Society (JRS-SBRTSG) database had PIC and were treated with SBRT for stage I NSCLC. Clinical background data of the patients are shown in Table 1. The median follow-up period for all patients was 23 months (range, 1–88 months). Figure 1 and Figure 2 show curves of overall survival (OS) and cause-specific survival (CSS), respectively. One, two, and three-year OS rates were 82.1% (95% confidence interval (CI), 76.9–87.3), 57.1% (CI, 49.8–64.5), and 42.6% (CI, 34.6–50.7), respectively. One, two, and three-year CSS were 95.9% (CI, 93.1–98.7), 78.9% (73.2–86.5), and 66.1% (57.0–75.1), respectively. Figure 3, Figure 4, Figure 5, Figure 6 and Figure 7 show OS according to gender (male versus female), T stage (T1 versus T2), histology (adenocarcinoma versus squamous cell carcinoma), % vital capacity (% VC) (≥70% versus <70%), and mean percentages of normal lung volume receiving more than 20 Gy (V20) (≥10% versus <10%). Results of univariate and multivariate analysis are shown in Table 2. Univariate analysis showed statistically significant differences in % VC (≥70% versus <70%) and V20 (≥10% versus <10%). Multivariate analysis showed statistically significant differences in performance status (0 and 1 versus 2–4), histology (adenocarcinoma versus squamous cell carcinoma), T stage (T1 versus T2), regular use of steroid administration before SBRT, and percentages of predicted forced expiratory volume in one second (FEV1.0 (%)) (≥70% versus <70%). Grade 3–5 and grade 5 RP occurred in 12.4% and 6.9% of patients, respectively. The incidence rates of severe RP according to the patient backgrounds are shown in Table 3. It has been found that V20 ≥ 10% was a major risk factor of severe RP.

## 3. Discussion

Incidence rates in previous reports of severe RP, with and without PIC after SBRT for lung cancer, are shown in Table 4 [3,4,5,6,7]. According to previous reports, radiation pneumonitis grade 3 and above were observed in 10–40% of patients who had PIC, and grade 5 was observed in more than 5% of these patients. Our study showed a similar incidence of severe toxicity in patients with PIC as previous retrospective reports have suggested [3,4,5], and the true rates may be higher in a prospective setting.

With respect to the relationship between RP and radiotherapy planning, Yamashita et al. [8] reported a high incidence of fatal RP (3 of 25 patients) after SBRT, which occurred in patients with poor respiratory function, interstitial pneumonitis (IP), and recurrence after surgery. In that study, fatal RP was reported as related to a large volume of lung parenchyma irradiated at high doses [8]. Timmerman et al. [9] reported a higher incidence of fatal RP after SBRT in patients whose tumors were located more centrally than peripherally. Hope et al. [10] and Matsuo et al. [6] found that fatal RP occurrence correlated with the volume of the higher dose region; however, Takeda et al. [11] claimed that severe RP post-SBRT for lung cancer was not related to lung dose.

Acute exacerbation of IP is considered to be a critical potential complication following surgery, chemotherapy, or radiotherapy for lung malignancies [12]. Takeda et al. reported acute exacerbation of subclinical idiopathic pulmonary fibrosis triggered by SBRT in a patient with slight focal honeycomb changes of the lung, and argued that attention must be paid to the presence of co-morbid IP, even if it is minimal [13].

Although the PIC is highly predictive of progressive RP, not all cases of PIC result in RP. Risk factors that can predict the development of severe RP include indicators of pulmonary function, dose-volume-fraction, serum biomarkers, such as KL-6, and single nucleotide polymorphism at rs1982073:T869C of the TGF beta 1 gene [14,15].

In regard to the incidence of severe RP, the fact that V20 more than 10% was the most outstanding risk factor in the current study indicated that a larger irradiated volume would also be a major risk factor. Patients with clinical T2 stage cancer were also one of subgroups with a high incidence of severe RP in the current study, which appeared to be due to a larger tumor size, resulting in higher V20. Higher dose-concentrated irradiation methods, such as deep-inspired breath-hold technique [16] or particle therapy, may be useful for sparing the background lung from high radiation doses [17].

There was a significant difference in OS according to FEV1.0 (%), which could be due to a lower pulmonary compliance, resulting from advanced lung fibrosis, which could then cause progressive respiratory failure.

In this study, the incidence of severe RP was high in the subgroup of patients with adenocarcinoma. The reason for this was unknown, but despite high incidence rates, OS of this group was generally good. One reason for the good prognosis of this group might be that some of these patients had early well-differentiated adenocarcinoma, such as adenocarcinoma in situ or minimally invasive adenocarcinoma, of which prognosis is generally very good [18].

Sato T et al. reported that the incidence of fatal acute exacerbation occurred after resection of lung cancer in patients with interstitial lung disease at a rate of 4.0%, and the surgical procedures, male sex, history of exacerbation, preoperative regular use of steroid use, serum sialylated carbohydrate antigen KL-6 levels, usual interstitial pneumonia appearance on computed tomography scan, and reduced percentage of predicted vital capacity were identified as risk factors of acute exacerbation [19]. The rate of fatal RP was lower than the incidence of fatal RP after SBRT according to our results (6.9%). However, more than three quarters of the patients were inoperable in our study, as shown in Table 1, and the background of the patients would be different in these two studies. Therefore, it is difficult to conclude which modality is safer as a radical treatment for stage I NSCLC patients with PIC.

Our study has some limitations. First, this is a retrospective study without unified criteria nor central review for the judgment of medical operability, PIC, and fatal RP. Secondly, calculation algorithms and prescription points were different among institutions; therefore, the dose-volume histogram of the normal lung was not standardized. Thirdly, disease progression may have had an additional effect on the cause of death in some patients with fatal RP. Therefore, it was difficult to demonstrate a true incidence of death related to fatal RP. Finally, we did not include other possible risk factors, such as detailed pulmonary function, dose-volume-fraction, and serum biomarkers, such as KL-6, in our analysis, because there were too many missing values. A future prospective multicenter study with a larger cohort is needed to validate the relationship between the PIC and severe radiation pneumonitis.

## 4. Materials and Methods

We used a retrospective multi-institutional study design, which was approved by the institutional review board of the University of Yamanashi Hospital (approval number 961, 19 July 2012) and opt outed in each of the institutions. The study was conducted in accordance with the Declaration of Helsinki. We organized a JRS-SBRTSG, and conducted studies of SBRT for primary or oligometastatic cancer in various organs, but primarily in the lung. The original JRS-SBRTSG database included data of more than 2000 patients for the study of SBRT for stage I NSCLC from 20 institutions. A board-certified thoracic radiologist in each institution examined the CT images of these patients to determine the presence of PIC and emphysema. At least one finding of ground glass opacities, reticular, small nodular, reticulonodular patterns, and honeycombing on thin-slice computed tomography (CT) images at time of SBRT were required to diagnose the PIC. TNM staging was decided according to the criteria of the Union for International Cancer Control (UICC) version 7. Medical operability for standard lobectomy with ipsilateral hilar and mediastinal lymph node dissection was judged according to each institution’s criteria.

SBRT techniques and dose calculation methods differed among institutions. The gross target volume (GTV) was delineated on CT images using a lung window-level setting. The clinical target volume (CTV) enlarged the GTV by 0–5 mm as judged by the individual radiation oncologist. The internal margin was calculated and set at 2–5 mm around the CTV according to the individual measurements for respiratory motion determined at each institution. The internal margin caused by respiratory motion was reduced by gating, tracking, the breath-hold technique, or abdominal compression. The planning target volume (PTV) comprised the CTV, a proper internal margin measured for each patient, and a 5-mm safety margin. The irradiated port marginally exceeded the PTV by 3–5 mm to secure the surface dose of the PTV. Dose calculation was mainly performed using the Clarkson or superposition algorithm with heterogeneity correction. A total dose of 40–70 Gy (mean, 58.7 Gy) was delivered mainly at the isocenter, but in some cases, at the periphery of the PTV in 4–10 fractions during 4 to 25 days with 6 MV X-rays. Typical dose/fractionation schedules were 48 Gy in four fractions. The median calculated biological effective dose (BED) was 105.8 Gy (range, 76.8–134.4 Gy) based on alpha/beta = 10.

OS, CSS, and prognostic factors for OS were analyzed. Examined candidates of prognostic factors were gender, age (≥80 years versus <80 years), performance status (0 and 1 versus 2–4), pathology (adenocarcinoma versus squamous cell carcinoma), T stage (T1 versus T2), medical operability, smoking history, pulmonary emphysema, regular use of steroid before SBRT, % VC (≥70% versus <70%), FEV1.0 (%) (≥70% versus <70%), BED (≥100 Gy versus <100 Gy), and V20 (≥10% versus <10%). Incidence rates of severe RP were calculated according to patient backgrounds.

Survival rates were calculated using the Kaplan-Meier method. Statistical differences were calculated with a log-rank test in univariate analysis and Cox proportional hazards model in multivariate analysis. Differences were judged as statistically significant for *p* < 0.05. All statistical analyses were performed using Statview software (SAS, Cary, NC, USA).

## 5. Conclusions

Fatal radiation pneumonitis was identified in 6.9% of 242 patients with PIC after SBRT for stage I lung cancer, and one, two, and three-year overall survival rates were 82.1%, 57.1%, and 42.6%, respectively. V20 more than 10% was a major risk factor of severe RP. All possible risk factors should be considered in determining patients that may be at risk for radiation pneumonitis. A prospective multicenter study is mandatory to validate true predictive factors.

## Figures and Tables

**Table 1:** Clinical backgrounds of the patients.

Total patients number	242
Gender	Male: 214, Female: 28
Age	55–92 (median: 77) y.o
Performance status (ECOG)	PS 0, 1, 2, 3, 4, NA * = 99, 114, 20, 2, 0, 7
Histology	squamous cell cancer: 92
	adenocarcinoma: 77
	unspecified non-small cell lung cancer: 18
	unproven: 55
Tumor size	3–50 mm (median: 27 mm)
T stage (7th UICC)	T1, T2 = 160, 82
Medical operability	operable:54, inoperable:175, NA *: 13
Smoking history	(+): 153, (−): 25, NA *: 64
Pulmonary emphysema	(+): 87, (−): 118, NA *: 37
Steroid administration before SBRT	(+): 20, (−): 174, NA *: 50
% vital capacity (% VC)	45.6–150.6% (mean: 91.9%)
FEV1.0% **	37.1–97.1% (mean: 67.9%)
SBRT dose	40–70 Gy in 4–10 fractions
V20 ***	1.1–21.7% (mean: 6.6%)

* NA: not assigned; ** FEV1.0%: percentage of predicted forced expiratory volume in one second; *** V20: rate of the volume irradiated with 20 Gy or more to the normal lung volume. (+): present; (−): absent.

**Table 2:** Univariate * and multivariate ** analysis for OS (*p* value).

Variables (If Significant, Left Was Better)	Univariate	Multivariate
Female vs. Male	0.26	0.03
Age <80 years vs. ≥80 years	0.69	0.88
Performance status 0,1 vs. 2,3,4	0.50	0.03
Adeno ca vs. Squamous cell ca	0.07	<0.01
T1 vs. T2	0.22	0.01
Medical operable vs inoperable	0.44	0.92
Smoking history (−) vs. (+)	0.33	0.10
Pulmonary emphysema (−) vs. (+)	0.24	0.13
Steroid administration before SBRT (−) vs. (+)	0.49	<0.01
% vital capacity (% VC) ≥70% vs. <70%	0.02	0.60
FEV1.0% *** ≥70% vs. <70%	0.98	<0.01
Biological effective dose ≥100 Gy vs. <100 Gy	0.13	0.90
V20 **** <10% vs. ≥10%	0.03	0.13

* Calculated using the Kaplan Meier’s method and log-rank test; ** Calculated using the Cox’s proportional hazard model; *** FEV1.0%: percentage of predicted forced expiratory volume in one second; **** V20: rate of the volume irradiated with 20 Gy or more to the normal lung volume. (+): present; (−): absent.

**Table 3:** Incidence of severe radiation pneumonitis according to patient backgrounds.

Patient Factors	Grade 3–5	Grade 5
Female vs. Male	12.1% vs. 15.4%	11.5% vs. 6.3%
Age <80 years vs. ≥80 years	13.2% vs. 11.2%	6.9% vs. 6.7%
Performance status 0,1 vs. 2,3,4	10.7% vs. 25.0%	6.8% vs. 5.0%
Adeno ca vs. Squamous cell ca	14.9% vs. 11.0%	9.5% vs. 5.5%
T1 vs. T2	11.6% vs. 10.7%	6.2% vs. 5.3%
Medical operable vs. inoperable	5.7% vs. 15.5%	5.7% vs. 7.7%
Smoking history (−) vs. (+)	13.0% vs. 12.8%	8.7% vs. 6.1%
Pulmonary emphysema (−) vs. (+)	11.5% vs. 16.7%	7.1% vs. 9.5%
Steroid administration before SBRT (−) vs. (+)	12.6% vs. 21.1%	7.8% vs. 5.3%
% vital capacity (%VC) ≥70% vs. <70%	5.3% vs. 12.0%	5.3% vs. 5.3%
FEV1.0% * ≥70% vs. <70%	9.9% vs. 13.4%	4.2% vs. 6.0%
Biological effective dose ≥100 Gy vs. <100 Gy	11.8% vs. 16.7%	6.9% vs. 6.7%
V20 ** < 10% vs. ≥10%	11.1% vs. 29.4%	6.0% vs. 28.6%

* FEV1.0%: percentage of predicted forced expiratory volume in one second; ** V20: rate of the volume irradiated with 20 Gy or more to the normal lung volume. (+): present; (−): absent.

**Table 4:** Frequency of radiation pneumonitis post SBRT for lung cancer according to presence of pulmonary interstitial change.

Pulmonary Interstitial Change	Author [Ref.]	Study Design	Patient Number	Dose/Fraction	Grade of Radiation Pneumonitis	Frequency	Risk Factor
No pulmonary interstitial change	Yamaguchi [3]	Retrospective	86	48 Gy/4 fr	4–5	0.00%	
	Ueki N [4]	Retrospective	137	48–60 Gy/4–8 fr	3–5	1.40%	
	Yoshitake [5]	Retrospective	242	48 Gy/4 fr	3	1.20%	
					4–5	0.00%	
	Matsuo Y [6]	Retrospective	74	48 Gy/4 fr	3	10.60%	V25
					4	1.90%	
	Nagata Y [7]	Prospective	104 (inoperable)	48 Gy/4 fr	3	6.20%	
			65 (operable)	48 Gy/4 fr	3	3.60%	
With pulmonary interstitial change	Yamaguchi [3]	Retrospective	16	48 Gy/4 fr	3	6.30%	V5–25, MLD
					4	6.30%	
					5	6.30%	
	Ueki N [4]	Retrospective	20	40–60 Gy/4–8 fr	3–5	10.00%	
	Yoshitake [5]	Retrospective	18	48 Gy/4 fr	3	16.70%	KL-6, V5, V10, MLD
					4–5	22.30%	
	This study	Retrospective	242	Various (mainly 48 Gy/4 fr)	3–5	12.40%	% VC, FEV1.0 (%), Squamous cell ca., V20, PS, T stage, steroid before SBRT
					5	6.90%	

Vx: rate of the volume irradiated with x Gy or more to the normal lung volume; MLD: mean dose of normal lung.
